# Person-centred care in interventions to limit weight gain in pregnant women with obesity - a systematic review

**DOI:** 10.1186/s12884-015-0463-x

**Published:** 2015-02-27

**Authors:** Ellinor K Olander, Marie Berg, Christine McCourt, Eric Carlström, Anna Dencker

**Affiliations:** Centre for Maternal and Child Health Research, School of Health Sciences, City University London, London, UK; Institute of Health and Care Sciences, Sahlgrenska Academy, University of Gothenburg, Gothenburg, Sweden; Centre for Person-Centred Care (GPCC), University of Gothenburg, Gothenburg, Sweden

**Keywords:** Person-centred care, Maternal obesity, Gestational weight gain, Intervention, Systematic review

## Abstract

**Background:**

Person-centred care, asserting that individuals are partners in their care, has been associated with care satisfaction but the value of using it to support women with obesity during pregnancy is unknown. Excessive gestational weight gain is associated with increased risks for both mother and baby and weight gain therefore is an important intervention target. The aims of this review was to 1) explore to what extent and in what manner interventions assessing weight in pregnant women with obesity use person-centred care and 2) assess if interventions including aspects of person-centred care are more effective at limiting weight gain than interventions not employing person-centred care.

**Methods:**

Ten databases were systematically searched in January 2014. Studies had to report an intervention offered to pregnant women with obesity and measure gestational weight gain to be included. All included studies were independently double coded to identify to what extent they included three defined aspects of person-centred care: 1) “initiate a partnership” including identifying the person’s circumstances and motivation; 2) “working the partnership” through sharing the decision-making regarding the planned action and 3) “safeguarding the partnership through documentation” of care preferences. Information on gestational weight gain, study quality and characteristics were also extracted.

**Results:**

Ten studies were included in the review, of which five were randomised controlled trials (RCT), and the remaining observational studies. Four interventions included aspects of person-centred care; two observational studies included both “initiating the partnership”, and “working the partnership”. One observational study included “initiating the partnership” and one RCT included “working the partnership”. No interventions included “safeguarding the partnership through documentation”. Whilst all studies with person-centred care aspects showed promising findings regarding limiting gestational weight gain, so did the interventions not including person-centred care aspects.

**Conclusions:**

The use of an identified person-centred care approach is presently limited in interventions targeting gestational weight gain in pregnant women with obesity. Hence to what extent person-centred care may improve health outcomes and care satisfaction in this population is currently unknown and more research is needed. That said, our findings suggest that use of routines incorporating person-centredness are feasible to include within these interventions.

**Electronic supplementary material:**

The online version of this article (doi:10.1186/s12884-015-0463-x) contains supplementary material, which is available to authorized users.

## Background

Pregnant women with obesity (body mass index, BMI ≥ 30) often report being negatively treated by health professionals and maternity services [1-3]. This adverse treatment may include not explaining the extra care women with obesity may need, discarding women’s often complex relationship with their body and weight or not treating women as independent individuals. A meta-synthesis summarising women with obesity’s experience with maternity services concluded that there is a depersonalisation of care due to medicalisation of the women’s pregnancies [1]. Women often perceive that the maternity services focus on the baby rather than the mother and her health [1]. Medicalising a woman’s pregnancy because of a raised BMI may lead to women perceiving their care to be impersonal [1], increase their discomfort [2] and feelings of guilt [4]. These pregnant women with obesity also report feeling judged and labelled by health professionals because of their weight [3-5]. Thus, it may be that pregnant women with obesity need to be given support in a personalised manner, tailored to the specific woman, incorporating her personal circumstances, capacities and goals [1,2,4].

Based on the negative reports from pregnant women with obesity [1-5], it seems imperative to assess to what extent healthcare interventions are adapted to the individual woman. Individually tailored care is something women want [4] and is also explicitly stated in a range of antenatal care guidelines (see for example [6,7]). Patient-centred care is widely advocated worldwide, with patients supporting its premise of a partnership approach to treatment [8]. Recently however, patient-centred care has been criticised for viewing individuals as patients and not persons [9]. Furthermore, the concept “patient” may not be relevant to all healthcare groups, such as pregnant women, as these women are not necessarily ill, simply pregnant. With this in mind, ‘person-centred care’ rather than ‘patient-centred care’ is a more applicable concept to use for a pregnant population.

A model of person-centred care was recently developed by Ekman and colleagues at the University of Gothenburg [9]. This model aims to look beyond the ‘patient’ to understand the person and is based on three aspects [9]. The first aspect, *initiating the partnership,* includes identifying the person’s narrative, including his/her individual account of their condition and subsequent impact on their life. This also includes eliciting what the individual wants from the care intervention, their goals and motivation. The second aspect, *working the partnership,* includes sharing of information and decision-making and focuses on developing a partnership to achieve commonly agreed goals. At the initial meeting, the health professionals and the individual should consider all aspects of care, taking into account care options that are suited to the person’s lifestyle, preferences, beliefs, values, and health issues. The third and last aspect, *safeguarding the partnership through documentation,* includes documenting the individual’s care preferences and treatment decision-making in patient records. The aim of this last aspect is to give legitimacy to the individual’s feelings, create a transparent relationship between individual and healthcare professional and build continuity in care [9].

Thus far, person-centred care research has been primarily focused on clinical populations (see for example [10]), and to our knowledge no research explicitly focused on person-centred approaches has been done in a pregnant population. Moreover, to our knowledge, no research to date has examined pregnant women with obesity and whether their care is using the three aspects of the person-centred care model as developed by Ekman et al. [9].

Hence, the primary aim of the current review was to explore the extent and in what manner interventions developed for pregnant women with obesity use person-centred care. A secondary aim was to assess if interventions that included aspects of person-centred care are more effective at limiting gestational weight gain than interventions not employing person-centred care aspects. To ensure all interventions were of clinical relevance they had to measure gestational weight gain as one of the outcomes. Gestational weight gain was chosen as a criterion for included interventions for several reasons. Firstly, pregnant women with obesity have been found to gain more weight than is recommended for their weight category [11]. Secondly, recent research suggests that pregnant women with obesity may need different support compared with women who start their pregnancy a healthy weight and person-centred care, involving partnership with the woman, may address this concern [12]. Thirdly, gestational weight gain has been associated with numerous risks for both mother and (indirectly) her baby. A large meta-analysis found that limiting gestational weight gain can reduce pre-eclampsia and shoulder dystocia [13]. Other studies have found that gaining an excessive amount of weight in pregnancy as defined by the American Institute of Medicine [14], may be associated with an increased risk of gestational diabetes, caesarean delivery [15], postpartum weight retention [16] and childhood obesity [11]. Thus reducing the risk of excessive gestational weight gain is of great clinical importance for pregnant women with obesity.

## Methods

A systematic literature review was conducted to identify interventions, which were then coded for aspects of person-centred care. Interventions are, in this paper, identified as programmes or strategies designed to produce behaviour changes or improve health status [17].

A systematic search of ten databases (AMED, CINAHL, Embase, Global Health, Maternity and Infant Care, Medline, PsycArticles, PsycINFO, Psychology and Behavioural Sciences, PubMed), the Cochrane Library and key research reviews [18-20] was conducted in January 2014. Scopus was used for forward searching. Search terms were developed in line with PICO (Population, Intervention, Comparison and Outcome) criteria and included words associated with pregnancy, obesity, interventions and weight (see Additional file 1 for an example of full electronic search strategy, please contact the first author for a copy of the full review protocol). No filter was applied concerning types of interventions or research design. Studies were included if they described an intervention or service which only recruited pregnant women with a BMI ≥ 30 (as assessed either pre-pregnancy or early on in pregnancy), and reported gestational weight gain as an outcome (either primary or secondary). Papers also had to be in English and in peer-reviewed journals. No studies were excluded due to poor study quality as the aim of this review was to identify the extent to which interventions used person-centred care. The search and identification of included studies were done by the first author, with all included studies agreed upon by all authors.

All included interventions were double coded (by EKO and AD) to assess to what extent they included aspects of person-centred care. These criteria were developed from work by Ekman et al. [9] and Olsson and colleagues [10], and included three aspects. Firstly, *initiate the partnership* where the woman’s narrative and life circumstances are identified. Secondly, *working the partnership,* where the woman (and sometimes family) develop a partnership with her health professional and share the decision making regarding her care. Lastly, *safeguarding the partnership through documentation,* where the woman’s preferences and values are documented. Interventions would score zero if no person-centred care aspects were identified with three being the maximum score if all aspects were identified. In case of disagreement, the other review authors (MB, CMcC and EC) coded the intervention independently before the study team discussed and reached a decision on the specific intervention. This happened in four cases, where a group deliberation decided the final outcome.

All interventions were also assessed using the Critical Appraisal Skills Programme [21] by two authors (EKO and AD) to assess study quality. The randomised controlled trials were assessed with the randomised controlled trial checklist and the remaining studies by the cohort checklist. Lastly, study population, intervention specifics and total gestational weight gain findings (in terms of means and differences between intervention and control group) were extracted from each study by the first author. Total weight gain was chosen as outcome measure due to all studies reporting this outcome (in contrast to number of women gaining an excessive amount of weight which was not reported by all studies). This was to assess whether interventions that included aspects of person-centred care may be more effective at limiting gestational weight gain than interventions not employing person-centred care aspects. Ethical approval was not required for this systematic review.

## Results

**Figure 1:**
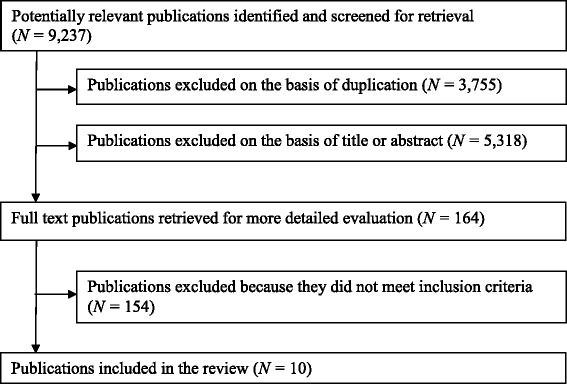
**Flowchart of review process.** Flowchart describing the number of articles retrieved, and included and excluded at each stage of the review process.

The search generated 9237 papers which when the exclusion criteria were applied, resulted in 10 papers [22-31] included in the review (see Figure 1). The main reasons for excluding papers were due to the studies not assessing gestational weight gain as an outcome or having an intervention that was available to all pregnant women irrespective of weight category. The forward search identified no new studies. See Table 1 for a summary of all included studies.

**Table 1:** **Summary of included studies**

**Authors, year (country)**	**Research design**	**Study aim** ^**1**^	**Intervention focus**	**Gestational weight gain measurement**	**Gestational weight gain results**
Baker, 2011 (England) [22]	Service development^1^	To develop a service to help prevent childhood obesity in the future by improving the health, eating habits and physical activity of pregnant women (p 633)	Physical activity and healthy eating	Not clear when baseline measure conducted, post measures at 38 weeks	Participants (N = 75) gained on average 7.3 kg (SD 5.7)
Claesson et al. 2008 (Sweden) [23]	Prospective case–control intervention study	To minimise obese women’s total weight gain during pregnancy to less than 7 kg (p 44)	Physical activity and healthy eating	Baseline ≤ 12 weeks, post measures at week of delivery or 1–2 weeks before delivery	Intervention group (N = 143) gained significantly less weight (8.7 kg, SD 5.51) compared to control group (N = 193; 11.3 kg, SD 5.80)
Ong et al. 2009 (Australia) [24]	Randomised controlled trial (intervention vs control group)	To assess the effect of exercise on glucose tolerance and aerobic fitness (p 419)	Physical activity	Measured at 18 and 28 weeks	No difference between intervention group (N = 6; 3.7 kg, SD 3.4) and control group (N = 6; 5.2 kg, SD 1.3).
Renault et al. 2014 (Denmark) [25]	Randomised controlled trial	To measure the effect on maternal gestational weight gain of a pedometer intervention with and without diet support (p 134.e2)	Physical activity (and in one intervention group physical activity and healthy eating)	Pre-pregnancy and post measure at 36–37 weeks	Both intervention groups gained less weight (physical activity group (N = 125; median 8.6 kg, range −9.6-34.1, physical activity and diet group N = 130; median 9.4 kg, range −3.4-28.2) than the control group (N = 134; median 10.9 kg, range −4.4-28.7). There was no difference between the intervention groups.
	Three groups; physical activity only, physical activity and diet, control				
Shirazian et al. 2010 (USA) [26]	Prospective historical cohort	To investigate whether a comprehensive lifestyle modification programme would be an effective way to limit weight gain during pregnancy and reduce associated obesity related complications (p 412)	Physical activity and healthy eating	First prenatal visit, not clear when post measure conducted	Intervention group (N = 21) gained significantly less weight (8.1 kg, SD 7.4) compared to matched historical control group (N = 20; 15.4 kg, SD 7.5).
Storck Lindholm et al. 2010 (Sweden) [27]	Prospective pilot study	To control weight gain through an intervention program with the primary aim of limiting maternal pregnancy weight gain to ≤ 6 kg (p 840)	Physical activity and healthy eating	Study entry (first trimester), no clear when post measure was conducted	Weight gain for group (N = 25) was 6.9 (SD 0.4) kg.
Thornton et al. 2009 (USA) [28]	Randomised clinical trial	To compare perinatal outcomes in the control versus intervention group (p 571)	Healthy eating	Pre-pregnancy, post measure was the last weight measurement before delivery	Intervention group (N = 116) gained significantly less weight (5.0 kg, SD 6.8) compared to control group (N = 116; 14.1 kg, SD 7.4 kg).
Vinter et al. 2011 (Denmark) [29]	Randomised controlled trial	To study the effects of a lifestyle intervention on gestational weight gain and obstetric outcomes (p 2502)	Physical activity and healthy eating	At study entry (<15 weeks) and at 35 weeks	Intervention group (N = 150) gained less weight (median 7.0, range 4.7-10.6 kg) compared to control group (N = 154; median 8.6, range 5.7-11.5 kg).
West, 2010 (England) [30]	Community service^1^	To create a service which encouraged a healthy weight gain (p 19)	Physical activity and healthy eating	No info provided	Participants (N = 291) gained on average 7.4 kg
Wolff et al. 2008 (Denmark) [31]	Randomised controlled trial	To assess if restriction of gestational weight gain can be achieved by dietary counselling (p 496)	Healthy eating	Pre-pregnancy and at delivery	Intervention group (N = 23) gained less weight (6.6 kg, SD 5.5) compared to control group (N = 27, 13.3 kg, SD 7.5).

^1^As described by study authors; SD = standard deviation.

### Study characteristics

The included studies came from Australia [24], Denmark [25,29,31], England [22,30], Sweden [23,27] and USA [26,28]. Restricting gestational weight gain was the main focus of seven interventions [23,25-27,29-31] with the others focused primarily on restricting childhood obesity [22], the effect of exercise on glucose tolerance and aerobic fitness [24] and other perinatal outcomes [28]. The number of women for whom gestational weight gain data was available for varied greatly, from a very small pilot study and service development studies to larger randomised controlled trials; thus sample sizes ranged from 12 [24] to 304 [29], with a mean of 170. On average, participants had a BMI of 35.1 when starting the intervention, were mostly White Caucasian, and 40% were first time mothers.

### Intervention characteristics

Intervention descriptions varied greatly, with some very well described and others unclear. In summary, the interventions were most often delivered in a healthcare setting [23,25,29] or in the participants’ homes [22,24]. All interventions were delivered by either an individual or a team of healthcare professionals, including midwives [23,27,30], dieticians [25,28,29,31], or healthcare personnel trained for the specific intervention [22]. Most studies focused on both physical activity and healthy eating behaviours [22,23,25-27,29,30]. Face-to-face was by far the most common delivery method, with only one study relying on telephone contact [25]. Lastly, the length of the interventions varied from a few sessions [30] to 26 weeks [25].

### Study quality

Five of the included studies were randomised trials [24,25,28,29,31], three were prospective studies [23,26,27] and two were community services [22,30]. The randomised trials were found to be of good quality, with clear benefits and clinically important outcomes (see Additional file 2 for trial CASP scores). The observational studies were of lower quality and it was sometimes uncertain whether the intervention and outcomes were measured accurately to minimise bias, and if all confounding factors were identified and accounted for (see Additional file 3 for cohort CASP scores). This was in particular the case for the two community services.

### Coding of person-centred care

Of the ten included studies, four interventions were found to include aspects of person-centred care (see Table 2) [22,23,30,31]. Or stated in a different manner – out of the 30 possible opportunities (10 studies, 3 aspects of person-centred care in each study) to incorporate person-centred care, only six (20%) instances were identified. Baker [22] and West [30] both included *initiating the partnership* and *working the partnership.* The health professionals in Baker’s intervention worked with the women to ‘identify small behaviour change goals around eating and activity’ (p635) and subsequently reviewed these goals at future meetings. When women struggled to keep active and eat healthily, the health professionals helped them find solutions [22]. West’s intervention included ‘initialise individualised care planning’ (p20) and was based on a supportive model with “ongoing support” and encouragement [30]. Claesson and colleagues [23] included *initiating the partnership* through conducting an interview with the woman early on in her pregnancy ‘to obtain any information relevant to her needs’ (p45) that may affect her motivation to change her behaviour (p45). Wolff et al. [31] included *working the partnership* through using women’s food records as ‘a tool to identify unhealthy eating patterns and give individualized suggestions for improvements’ (p496). No studies were found to include *safeguarding the partnership through documentation*.

**Table 2:** **Definitions and examples of person-centred care (PCC) and scores for all included studies**

	**Initiate the partnership**	**Working the partnership**	**Safeguarding the partnership through documentation**	
Definition	This includes identifying the patient narrative, including the individual’s personal account of his/her illness, symptoms, and their impact on her/his life. As well as what they may want from the intervention, their goals and motivation.	This includes sharing of information, shared deliberation, and shared decision making and focuses on developing a partnership to achieve commonly agreed goals.	This includes documenting patient preferences, beliefs, and values, as well as involvement in care and treatment decision-making in patient records.	
Example	Often done through interview/focus group or self-assessment at the beginning of intervention/care.	This may occur through a developed action plan by health professional(s) and woman, or shared decision making in setting goals with woman. Also includes collaborating on issues such as care pathway.	This may include a workbook where the woman describes her goals, enablers and barriers or documenting a discharge plan.	
**Author and year**				**Total PCC score**
Baker, 2011 [22]	Yes	Yes	No	2
Claesson et al. 2008 [23]	Yes	No	No	1
Ong et al. 2009 [24]	No	No	No	0
Renault et al. 2014 [25]	No	No	No	0
Shirazian et al. 2010 [26]	No	No	No	0
Storck Lindholm et al. 2010 [27]	No	No	No	0
Thornton et al. 2009 [28]	No	No	No	0
Vinter et al. 2011 [29]	No	No	No	0
West, 2010 [30]	Yes	Yes	No	2
Wolff et al. 2008 [31]	No	Yes	No	1
Total score	3	3	0	6

Note: Definitions and examples based on Ekman et al. and Olsson et al. [9,10]. Interventions were scored ‘1’ for if the person-centred care aspect was present and ‘0’ if absent.

### Gestational weight gain

The four interventions that were found to include one or two parts of person-centred care reported favourable gestational weight gain findings (see Table 1) [22,23,30,31]. Baker reported that their participants gained on average 7.3 kg [22], which is similar to West’s study where women gained on average 7.6 kg [30]. Claesson et al. compared their intervention group to a case–control group and found that their intervention group gained less weight than their control group (8.7 kg versus 11.3 kg) [23]. Lastly, Wolff and colleagues reported that their intervention group gained less gestational weight compared to their control group (6.6 kg versus 13.3 kg) [31].

The interventions that were not found to include any aspects of person-centred care also reported positive findings regarding gestational weight gain, outlined in Table 1 [25-29]. Only Ong et al. [24] did not find a significant difference in gestational weight gain between groups.

## Discussion

This review has explored to what extent aspects of person-centred care as defined by Ekman et al. [9] are included in interventions developed to limit the gestational weight gain of pregnant women with obesity. Four of the ten included studies were found to include the defined person-centred care aspects, with no study including all aspects. More specifically, of the 30 opportunities to include person-centred care, only six were identified. This highlights how seldom person-centred care as conceptualised by Ekman et al. [9] is reported to be used in these types of interventions. Notably the two studies that included two aspects of person-centred care each were community services developed primarily for the local population [22,30], not for research purposes. Two research studies included one aspect of person-centred care each [23,31]. None of the included studies mentioned being based upon or influenced by person-centred care, hence the person-centred care aspects had to be inferred in the review process from the intervention descriptions.

There were no obvious differences between the interventions that included or did not include person-centred care aspects in terms of gestational weight gain. All studies reported results in line with the Institute of Medicine guidelines on gestational weight gain [14], although not all studies stated at what time points they measured weight gain [22,26,27,30]. One study reported no differences between intervention and control group most likely due to the low sample size (N = 6 in each group) and the fact that weight was only measured between 18 to 28 weeks gestation [24]. Our findings support those of a prior meta-analysis indicating that interventions can limit gestational weight gain [13]. That said, a recent large randomised controlled trial found no difference in overweight and obese women’s gestational weight gain [32]. Clearly, more research is needed into identifying what makes some interventions successful and others not.

### Strengths and limitations

As is often the case with research, some limitations need to be noted. Firstly, the included ten studies varied greatly in terms of study design and focus, methodology and assessment of gestational weight gain. This has also been found in previous reviews [20] and limits the conclusions that can be drawn. That said, for this exploratory review, it is a considered strength that all study designs were included, as if only randomised controlled trials had been included, five studies would have been missed. Of those five, three included person-centred care aspects.

Secondly, a difficulty regarding the studies was their varied intervention description that made coding person-centred care aspects sometimes challenging. Again, this has been reported by other reviews relying on intervention coding [19] and in particular it was sometimes difficult to distinguish between the intervention and the study measures. To ensure reliability, the person-centred care and study design coding was independently double coded by two researchers (EKO and AD, a maternal health researcher and a midwife, both with extensive research experience). Any disagreements were discussed and decisions agreed by all review authors. Despite this, it is possible that some interventions have wrongfully been identified as not incorporating elements of person-centred care. Future research needs to ensure that interventions are clearly described using precise language, to simplify intervention coding and comparison.

It could be considered a limitation that only studies that recruited pregnant women with obesity were included in this review. Several studies that assessed gestational weight gain in pregnant women of all pre-pregnancy weight categories were excluded. Whilst we decided to focus on pregnant women with obesity due to their common reports of care dissatisfaction [1,2], it is likely that all pregnant women will benefit from person-centred care. More research is needed to assess whether the same pattern is found in interventions focused on pregnant women with a healthy or overweight weight status.

### The inclusion of person-centred care aspects

There are several important arguments for why person-centred care should be included into maternity care and interventions targeting pregnant women with obesity. First of all, pregnant women expect care that is tailored to them as individuals, not their weight status [4]. Research also suggests that tailored health behaviour interventions may be more effective in changing behaviours compared to non-tailored interventions [33,34]. Furthermore, the need to provide woman-focused care is in care guidelines worldwide [6,7], reflecting the research that indicates the value of this from the woman’s emotional as well as physical wellbeing perspective. It is thus disappointing that there has not been more focus on assessing maternity services from a person-centred care perspective. Whilst it is possible that the women’s usual care was based on person-centred care, none of the interventions explicitly stated they were based on person-centred care. This may be due to it being a fairly new concept, as nine of the ten included studies were published before or during the same year as Ekman et al’s paper [9]. However, the need for more women-centred approaches to maternity care has been discussed over a longer period.

The review process was not confined to the specific term ‘person-centred’ and used some inference to identify the three aspects defined by Ekman et al. [9]. Whilst not explicitly stating that their interventions were based on person-centred care aspects, four interventions were identified as including some aspects. Three interventions included initiating the partnership [22,23,30]. These interventions included features such as individualised care planning [30] and encouraging the women to identify their own behavioural goals [22]. Claesson et al. [23]. started their intervention with a midwife interviewing the participating woman to identify her individual needs that may influence her motivation towards behaviour change In a separate study, the participants in Claesson et al’s [35] intervention reported enjoying these motivational meetings with the midwife. Taken together, these interventions indicate that aspects of person-centred care are feasible to include into maternity services. Moreover this could be an important part of care considering how many factors may influence a woman’s gestational weight gain [36] and that women may not attend services if they feel they do not meet their needs [37].

Two of the interventions that included initiating the partnership also included working the partnership. After Baker’s [22] participants had chosen their own behaviour goals these goals were reviewed. If the women struggled to keep active and/or eat healthily, the health professionals helped them find individualised solutions. Similarly, West [30] offered her participants ongoing support based on a supportive model of care. Wolff et al. [31] also included working the partnership through using women’s food records to identify unhealthy eating and provide individualised suggestions for improvements. Again, this shows that person-centred care can be included into interventions for pregnant women with obesity. It also suggests that working the partnership could facilitate continuity of care, which has been associated with maternal care satisfaction [38]. Lastly, it is curious to note that it was two service evaluations which included the most person-centred care aspects (two) [22,30]. Whilst the reason for this is unclear, it may be that their focus was on tailored support for pregnant women with obesity and not testing an intervention that needs to be standardised. Their papers’ focus on the service development and less on their outcomes (i.e. neither paper provides details on time-points for gestational weight gain measures) would support this explanation.

No interventions were found to include the person-centred care aspect safeguarding the partnership through documentation. This aspect is described by Ekman et al. as documenting the individuals care preferences and beliefs [9]. It is somewhat surprising that this aspect was not identified in any of the studies as maternity care often recommend women working together with their healthcare professional to develop and document a birth plan [6]. More research is needed to include safeguarding the partnership through documentation in interventions for pregnant women with obesity.

### Practical implications and future research

This review suggests that aspects of person-centred care have not yet been fully included in interventions promoting limited weight gain through a changed lifestyle in pregnant women with obesity. According to a qualitative study, utilising a person-centred care approach may decrease the number of pregnant women with obesity who are dissatisfied with their maternity care [2]. The number of women who start their pregnancy obese is increasing [39-41], and maternal obesity is argued to be one of the great challenges for maternity care now and in the future [42]. Hence, healthcare professionals need to be prepared to support pregnant women with obesity in a suitable manner.

Importantly, supporting pregnant women with weight management entails more than advising a woman to eat healthily and keep physically active [43]. It also includes a willingness to understand the causes of the woman’s weight and providing them with care without bias [7]. Thus, healthcare professionals need to be given time to create a care partnership with pregnant women and together identify the factors that may influence her weight gain (see Hill et al. for a conceptual model of factors that may influence gestational weight gain [36]). Health care professionals may also need to be given support and training to help them incorporate person-centred aspects into their care.

Healthcare professionals play an important role in supporting pregnant women with obesity. Recent research suggests that women want weight management support from their midwife [44] and that they rely on their healthcare professional to inform them of risks associated with their pregnancy [3,45]. Providing care for pregnant women with obesity in line with the person-centred care ethos conceptualised by Ekman and colleagues would include forming a partnership with the woman concerning her preferences and care [9]. This would include establishing the woman’s specific needs and circumstances related to her pregnancy. It is likely that her weight management support needs depend on her current behaviour, psychological factors such as mental health, social-contextual factors such as social support and knowledge regarding gestational weight gain [36]. It would also include sharing information with the ultimate goal of aiding the woman to achieve her goals towards healthy behaviour change in pregnancy. For example, women may be highly motivated towards healthy eating and physical activity in pregnancy [46] but also face barriers towards these behaviours such as lack of time and confidence in their ability to change their behaviour [47]. The person centred care developed by Ekman et al. would also include sharing the deliberation and decision-making regarding the woman’s care and documenting this decision-making as well as the woman’s preferences. For example, this could be done through deciding appropriate weight gain goals with the woman (based on her circumstances and not solely the national guidelines) and documenting this in her maternity notes. This review found no interventions which were identified as including all three aspects of person-centred care and future research is thus needed. As well as testing interventions that include aspects of person-centred care [9], further research is also needed to study the utility of the three aspects of person-centred care.

## Conclusions

To our knowledge, this is the first review exploring to what extent and in what manner aspects of person-centred care, defined as including three essential aspects [9] are included in interventions aimed at limiting pregnant women with obesity’s gestational weight gain. Less than half of the eligible interventions were identified as including aspects of person-centred care and there were no obvious gestational weight gain differences between the interventions that included aspects of person-centred care compared to those that did not. That said, our findings suggest that using a model of person-centred care is feasible to include into health promotion interventions and services for pregnant women with obesity. Research is now needed testing the merits of incorporating such a model of person-centred care in maternal health interventions and services. To include the person-centred care ethos in maternity services would be in line with most national guidelines worldwide [6,7].

## Additional files

Additional file 1:
**AMED search 24th of January, 2014.**
Additional file 2:
**CASP scores for trials.**
Additional file 3:
**CASP scores for cohort studies.**
