# Supplementary material for: Person-centred care in interventions to limit weight gain in pregnant women with obesity - a systematic review
Source: BMC Pregnancy Childbirth. 2015 Feb 27;15:50. doi: 10.1186/s12884-015-0463-x (PMC4350295; doi:10.1186/s12884-015-0463-x)
Supplement: Additional file 1: — AMED search 24th of January, 2014. [file 12884_2015_463_MOESM1_ESM.docx]

Additional file 1 - AMED search 24^th^ of January, 2014

1. Pregnancy
2. Obesity
3. Body mass index
4. 2 or 3
5. Program evaluation or patient education
6. Delivery of health care of health services
7. Comparative study OR randomized controlled trials or Clinical trials
8. 5 or 6 or 7
9. Obesity
10. Pregn*(abstract) OR pregn* (title) OR antenatal*(abstract) OR antenatal* (title) OR perinata* (abstract) OR perinata* OR gestatio* (abstract) OR gestatio* (title)
11. Intervention*(abstract) OR intervention(title) or Progra*(abstract) or program*(title), servic* (abstract) OR servic*(title) Or tria* (abstract) OR tria* (title)
12. Weigh* (abstract) OR weigh* (title)
13. Obes* (abstract) OR obes* (title) OR BMI (abstract) OR BMI (title)
14. 1 OR 10
15. 4 OR 13
16. 8 OR 11
17. 9 OR 12
18. 14 AND 15 AND 16 AND 17
