# Supplementary material for: Person-centred care in interventions to limit weight gain in pregnant women with obesity - a systematic review
Source: BMC Pregnancy Childbirth. 2015 Feb 27;15:50. doi: 10.1186/s12884-015-0463-x (PMC4350295; doi:10.1186/s12884-015-0463-x)
Supplement: Additional file 2: — CASP scores for trials. [file 12884_2015_463_MOESM2_ESM.docx]

Additional file 2 - CASP scores for trials

| **Author** | **Q1 Did the trial address a clearly focused issue?** | **Q2 Was the assignment of patients to treatments randomised?** | **Q3 Were all of the patients who entered the trial properly accounted for at its conclusion?** | **Q4 Were patients, health workers and study personnel 'blind' to treatment?** | **Q5 Were the groups similar at the start of the trial?** |
| --- | --- | --- | --- | --- | --- |
| Ong et al, 2009 [22] | Yes | Yes | Yes | No | Yes |
| Renault et al, 2014 [23] | Yes | Yes | Yes | No | Yes |
| Thornton et al, 2009 [26] | Yes | Yes | Yes | No | Yes |
| Vinter et al, 2011 [27] | Yes | Yes | Yes | No | Yes |
| Wolff et al, 2008 [29] | Yes | Yes | Yes | No | Yes |

| **Author** | **Q6 Aside from the experimental intervention, were the groups treated equally?** | **Q7 How large was the treatment effect?** | **Q8 How precise was the estimate of the treatment effect?** | **Q9 Can the results be applied in your context/or the local population?** | **Q10 Were all clinically important outcomes considered?** | **Q11 Are the benefits worth the harms and costs?** |
| --- | --- | --- | --- | --- | --- | --- |
| Ong et al, 2009 [22] | Yes | No information provided | Precise | Yes | Yes | Most likely |
| Renault et al, 2014 [23] | Yes | No information provided | Precise | Yes | Yes | Yes |
| Thornton et al, 2009 [26] | Yes | No information provided | Precise | Yes | Yes | Yes |
| Vinter et al, 2011 [27] | Yes | No information provided | Precise | Yes | Yes | Yes |
| Wolff et al, 2008 [29] | Yes | No information provided | Precise | Yes | Yes | Yes |

For question 4 it is impossible to blind individuals to this type of study. For question 5, we examined differences in weight or BMI only.
