# Supplementary material for: Person-centred care in interventions to limit weight gain in pregnant women with obesity - a systematic review
Source: BMC Pregnancy Childbirth. 2015 Feb 27;15:50. doi: 10.1186/s12884-015-0463-x (PMC4350295; doi:10.1186/s12884-015-0463-x)
Supplement: Additional file 3: — CASP scores for cohort studies. [file 12884_2015_463_MOESM3_ESM.docx]

| Author | Q1 Did the trial address a clearly focused issue? | Q2 Was the cohort recruited in an acceptable way? | Q3 Was the exposure accurately measured to minimise bias? | Q4 Was the outcome accurately measured to minimise bias? | Q5a Have the authors identified all important confounding factors? Were the groups similar at the start of the trial? | Q5b Have the authors taken account of the confounding factors in the design and/or analysis? | Q6a Was the follow-up of subjects complete enough? |
| --- | --- | --- | --- | --- | --- | --- | --- |
| Baker, 2011 [20] | Yes | No | Cannot tell | Cannot tell | No | No | No |
| Claesson et al, 2008 [21] | Yes | Yes | Yes | Yes | Yes | Yes | Yes |
| Shirazian et al, 2009 [24] | Yes | Yes | Yes | Yes | No | Cannot tell | Yes |
| Storck Lindholm et al, 2010 [25] | Yes | Yes | Yes | Yes | No | No | Yes |
| West, 2010 [28] | Yes | No | Cannot tell | Cannot tell | No | Cannot tell | No |

Additional file 3 – CASP scores for cohort studies

| Author | Q6b Was the follow up long enough? | Q7 What are the results of this study? | Q8 How precise are the results? | Q9 Do you believe the results? | Q10 Can the results be applied to the local population? | Q11 Do the results of this study fit with other available evidence? | Q12 What are the implications of this study for practice? |
| --- | --- | --- | --- | --- | --- | --- | --- |
| Baker, 2011 [20] | Yes | Women gained on average 7.3 kg | Precise | Yes | Yes | Yes | Support regarding healthy eating and physical activity can help women gain healthy weight in pregnancy |
| Claesson et al, 2008 [21] | Yes | Intervention group gained less weight than control group | Precise | Yes | Yes | Yes | Lifestyle support can help women gain a healthy weight in pregnancy |
| Shirazian et al, 2009 [24] | Yes | Intervention group gained less weight than control group | Precise | Yes | Yes | Yes | Lifestyle intervention can help women gain a healthy weight in pregnancy |
| Storck Lindholm et al, 2010 [25] | Yes | Majority of women kept within weight goal | Precise | Yes | Yes | Yes | Lifestyle interventions can help women gain a healthy weight in pregnancy |
| West, 2010 [28] | Yes | Women gained on average 7.6 kg | Precise | Yes | Yes | Yes | Care pathways can be developed and help women limit their weight gain in pregnancy |

Additional file 3 – CASP scores for cohort studies continued
